# Thematic clustering of text documents using an EM-based approach

**DOI:** 10.1186/2041-1480-3-S3-S6

**Published:** 2012-10-05

**Authors:** Sun Kim, W John Wilbur

**Affiliations:** 1 National Center for Biotechnology Information, National Library of Medicine, National Institutes of Health, Bethesda, MD 20894, USA

## Abstract

Clustering textual contents is an important step in mining useful information on the web or other text-based resources. The common task in text clustering is to handle text in a multi-dimensional space, and to partition documents into groups, where each group contains documents that are similar to each other. However, this strategy lacks a comprehensive view for humans in general since it cannot explain the main subject of each cluster. Utilizing semantic information can solve this problem, but it needs a well-defined ontology or pre-labeled gold standard set. In this paper, we present a thematic clustering algorithm for text documents. Given text, subject terms are extracted and used for clustering documents in a probabilistic framework. An EM approach is used to ensure documents are assigned to correct subjects, hence it converges to a locally optimal solution. The proposed method is distinctive because its results are sufficiently explanatory for human understanding as well as efficient for clustering performance. The experimental results show that the proposed method provides a competitive performance compared to other state-of-the-art approaches. We also show that the extracted themes from the MEDLINE^® ^dataset represent the subjects of clusters reasonably well.

## Background

The text clustering task is to arrange a set of text documents into clusters such that the documents within each cluster are similar to each other. In text clustering, text is normally mapped to a vector space, i.e., documents are represented as bag-of-words, and each document becomes a feature vector using a weighting scheme. Clustering is then performed by measuring the distance between feature vectors. This common strategy is simple and clear to understand. However, the vector space mapping raises problems: the high dimensionality of the feature space and data sparsity [1]. Another important issue in this setup is the lack of humanly understandable results. To overcome the *curse of dimensionality *various techniques such as random indexing [2], subspace clustering [3] and topic modeling [4,5] can be utilized. Topic modeling is also a possible candidate for humanly understandable results since it extracts words attached to each topic.

MEDLINE [6], the largest biomedical literature database, consists of more than 20 million citations, and its volume is increasing rapidly. A partial solution for this overload issue is document clustering and summarization in a humanly understandable form. This can provide condensed text information from similar documents in a large repository. The vector space model may provide high-performance clustering in general, but an additional process is required to get keywords or a summarized description from the obtained clusters. Topic models are based upon the idea that documents are mixtures of topics, where a topic is a probability distribution over words [7]. A list of keywords that represent a topic can be obtained using these approaches. However, extracted topics should be followed by a clustering procedure since topic models are basically not designed for clustering.

An ontology is a conceptual framework which defines entities and their hierarchical relationships. Ontologies can be used to represent documents at a semantic level [8,9], but this concept-based model needs a well-defined database or a gold standard set for mapping words to pre-defined concepts. Key phrase-based approaches were also proposed for text clustering [10,11]. Key phrase extraction constructs a human-friendly feature set. Therefore, it can provide brief summaries of large documents. Highlighting key phrases in text also may increase readability. However, the current methods do not provide an integrated solution for phrase selection and clustering. Hofmann [12] presented the cluster-abstraction model for text data. While this model integrates clustering and keyword selection, it rather focuses on learning topic hierarchies.

Other works similar to our approach are ASI (Adaptive Subspace Iteration) [13] and SKWIC (Simultaneous Keyword Identification and Clustering of text documents) [14]. Both approaches perform document clustering and cluster-dependent keyword identification simultaneously. But, SKWIC has an additional feature in that it learns weights of keywords in addition to keyword identification. Nonetheless, SKWIC can only produce a fixed number of clusters. ASI is also computationally expensive because this algorithm heavily depends on matrix operations.

In this paper, we present a thematic clustering algorithm for text documents. Themes are initially unknown, however we assume that themes can be described by subject terms (keywords) in given text. In a probabilistic framework, subject terms are selectively chosen and used for partitioning document sets. An EM approach forces documents to be assigned to correct themes, hence it converges to a locally optimal solution. Unlike topic modeling, the proposed method integrates keyword selection and document clustering. The number of clusters is dynamically adjusted by probabilistic evidence from documents. The proposed approach is also reasonably fast compared to topic modeling approaches. Hence, the clustering results from our thematic clustering are sufficiently explanatory for human understanding as well as efficient for clustering performance. The experimental results on 20-Newsgroup [15] and MEDLINE show that the proposed method produces a set of well-defined topics with a competitive performance compared to other state-of-the-art algorithms.

## Methods

The proposed method is slightly different from common clustering approaches. The main focus of the thematic clustering algorithm is to find a text description, i.e., keywords, of the subjects that occur in a document set. In this regard, finding clusters is rather a secondary, but necessary feature to gather documents describing specific themes. Here, we first explain the theme generation framework introduced in [16], and extend the work for thematic clustering.

The theme generation framework is the EM formulation for thematic analysis of text documents, and consists of an E-step (expectation step) and an M-step (maximization step). Let *D *be a document set and let *T *be the set of index terms appearing in *D*. These index terms are a user-defined set, e.g., unigrams and bigrams. *R *denotes a relation between elements of *T *and *D*, i.e., *R *⊆ *T × D*. We define *tRd *to mean *t *∈ *d*. A theme is a particular subject that is discussed by a subset of documents in *D *using a subset of terms in *T*. Hence, a theme is defined as non-empty sets *U *⊆ *T *and *V *⊆ *D*, where all the elements of *U *have a high probability of occurring in all the element of *V*.

For a theme described by *U *and *V*, there are observed data *R *and missing data {*z_d_*}_*d*∈*D*_. The missing data *z_d _*is an indicator variable, i.e., *z_d _*= 1 when *d *∈ *V *and *z_d _*= 0 when *d *∉ *V*. The parameters are

(1) $$\Theta =U\left(||U||=n_{U}\right),{\left\{p_{t},q_{t}\right\}}_{t\in U},{\left\{r_{t}\right\}}_{t\in T},$$

where *n_U _*is the size of the set *U*, i.e., the number of subject terms. For any *t *∈ *U*, *p_t _*is the probability that for any *d *∈ *V*, *tRd*. *q_t _*is the probability that for any *d *∈ *D *- *V*, *tRd*. For any *t *∈ *T*, *r_t _*is the probability that for any *d *∈ *D*, *tRd*. In addition, {*pr_d_*}_*d*∈*D *_is defined as the set of prior probabilities that the elements *d *belong to *V*.

To facilitate mathematical formulation, we define two indicator variables *u_t _*and *δ_td_*. *u_t _*= 1 if *t *∈ *U *and *u_t _*= 0, otherwise.*δ_td _*= 1 if *tRd *and *δ_td _= *0, otherwise. We also assume that all relations *tRd *are independent of each other.

Now, our goal is to obtain the highest probability

(2) $$p\left(R,\;\left\{z_{d}\right\}|\Theta \right)=p\left(R|\left\{z_{d}\right\},\;\Theta \right)p\left(\left\{z_{d}\right\}|\Theta \right).$$

Computing from the right side in (2), we obtain

(3) $$p\left(\left\{z_{d}\right\}|\Theta \right)=\underset{d\in D}{\prod }pr_{d}^{z_{d}}{\left(1-pr_{d}\right)}^{1-z_{d}}.$$

(4) $$\begin{array}{llll} p\left(R|\left\{z_{d}\right\},\Theta \right)= & \underset{t,d}{\prod }\{[{\left(p_{t}^{\delta_{td}}{\left(1-p_{t}\right)}^{1-\delta_{td}}\right)}^{u_{t}}\; & & \;\\ & {\left(q_{t}^{\delta_{td}}{\left(1-q_{t}\right)}^{1-\delta_{td}}\right)}^{1-u_{t}}]z_{d}\; & & \;\\ & {\left(r_{t}^{\delta_{td}}{\left(1-r_{t}\right)}^{1-\delta_{td}}\right)}^{1-z_{d}}\}.\; & & \;\\ & \; & \end{array}$$

Next, as the E-step of the algorithm, Eqn 2 can be rewritten by taking the expectation of its logarithm, i.e.,

(5) $$\begin{array}{l} E\left(\text{ln}P\left(R|\left\{z_{d}\right\},\;\Theta \right)\right)=\\ \underset{t}{\sum }u_{t}\underset{d}{\sum }pz_{d}\left(\delta_{td}\text{ln}p_{t}+\left(1-\delta_{td}\right)\text{ln}\left(1-p_{t}\right)\right)+\\ \underset{t}{\sum }u_{t}\underset{d}{\sum }\left(1-pz_{d}\right)\left(\delta_{td}\text{ln}q_{t}+\left(1-\delta_{td}\right)\text{ln}\left(1-q_{t}\right)\right)+\\ \underset{t}{\sum }\left(1-u_{t}\right)\underset{d}{\sum }\delta_{td}\text{ln}r_{t}+\left(1-\delta_{td}\right)\text{ln}\left(1-r_{t}\right). \end{array}$$

In order to complete this calculation, it is necessary to compute *pz_d _*= *p*(*z_d _*= 1*|R*, Θ). By using Bayes' theorem, *pz_d _*is presented in a simpler form [16] as follows:

(6) $$pz_{d}=\frac{1}{1+\text{exp}\left(-score_{d}+C\right)},$$

where

(7) $$C=\underset{t\in U}{\sum }\text{ln}\left(\frac{1-p_{t}}{1-q_{t}}\right),$$

(8) $$score_{d}=\text{ln}\;\;\left(\frac{pr_{d}}{1-pr_{d}}\right)\;+\;\underset{t\in U}{\sum }\delta_{td}\text{ln}\;\left(\;\frac{p_{t}\left(1-q_{t}\right)}{q_{t}\left(1-p_{t}\right)}\right).$$

The M-step is to carry out the maximization of (5) over Θ. To achieve this, we may choose the values of *p_t_*, *q_t_*, and *r_t_*. By doing this, the individual sums on the right in (5) must be maximal for *p_t _*and *q_t _*when *u_t _*= 1 and for *r_t _*when *u_t _*= 0. Therefore,

(9) $$p_{t}=\;\;\frac{\sum_{d}\delta_{td}pz_{d}}{\sum_{d}pz_{d}},$$

(10) $$q_{t}=\;\;\frac{\sum_{d}\delta_{td}\left(1-pz_{d}\right)}{\sum_{d}\left(1-pz_{d}\right)},$$

(11) $$r_{t}=\;\;\frac{n_{t}}{N}.$$

where $n_{t}=\sum_{d}\delta_{td}$ and *N *= |*D*|.

For each *t*, we define a quantity *α *which is the difference between the contribution coming from *t *in the sum (5) depending on whether *u_t _*= 1 or *u_t _*= 0.

(12) $$\begin{array}{llll} \alpha_{t}= & n_{st}\text{ln}\left(\frac{p_{t}}{q_{t}}\right)+\left(n_{s}-n_{st}\right)\text{ln}\left(\frac{1-p_{t}}{1-q_{t}}\right)+\; & & \;\\ & \left(n_{t}-n_{st}\right)\text{ln}\left(\frac{q_{t}}{r_{t}}\right)+\; & & \;\\ & \left(N-n_{t}-n_{s}+n_{st}\right)\text{ln}\left(\frac{1-q_{t}}{1-r_{t}}\right),\; & & \;\\ & \; & \end{array}$$

where $n_{s}=\sum_{d}pz_{d}$ and $n_{st}=\sum_{d}\delta_{td}pz_{d}.$

Finally the maximization is completed by choosing the *n_U _*largest *α_t_*'s and setting *u_t _*= 1 for each of them and *u_t _*= 0 for all others.

This EM approach formulates how to choose the best subject terms from a set of documents. However, a document set may have multiple themes in general, hence this thematic analysis should be extended for multi-cluster approaches. Note that *pz_d _*is the probability that the document *d *includes a specific theme. Assuming that a document has a unique most prominent theme, this multi-cluster problem can be easily handled by assigning a document to the theme that has the highest *pz_d_*.

Table 1 denotes the procedure for our theme-based clustering algorithm. Given the input parameters, the initial number of clusters *K*, the number *n_U_*, and the set of prior probabilities {*pr_d_*}_*d*∈*D*_, a series of random clusters are first generated. The rest of the theme analysis steps are all performed independently except for assigning clusters. For each cluster *V_i_*, the probabilities {*pz_d_*}_*d*∈*D *_are estimated by using Eqn 6. Then for each document, it is assigned to the cluster for which *pz_d _*is the greatest. Step 4 through Step 7 are straightforward. To obtain the highest *n_U _α_t_*'s the parameters *p_t_*, *q_t_*, and *r_t _*are calculated. The termination condition of this algorithm is whether any change occurs in clusters. If there are no changes for all quantities, it is assumed that the current solution is converged. Another way for testing convergence is observing *C *in Eqn 7. If converged, the value of *C *will have the identical value on following iterations.

**Table 1:** The thematic clustering algorithm

Given *K *initial clusters, the number *n_U_*, and the set of prior probabilities {*pr_d_*}_*d*∈*D*_,
1. Create a random partition ${\left\{V_{i}\right\}}_{i=1}^{K}$ of *D *with corresponding relations ${\left\{R_{i}\right\}}_{i=1}^{K}$.
2. Compute *p_t_*, *q_t_*, and *r_t _*for *V_i_*.
3. Compute *α_t _*for *V_i_*.
4. For each cluster, select the *n_U _*points for which *α_t _*is the greatest to define the set *U *and the indicator values {*u_t_*}_*t*∈*T*_.
5. Compute the probabilities {*pz_d_*}_*d*∈*D *_for each cluster *V_i_*.
6. For all *d*, assign a document to the cluster in which the document has the highest probability.
7. Test for convergence. Terminate if converged.
8. For a subset $D_{s}\subset D_{V_{i}}$, where the documents in *D_s _*has the lowest 1% {*pz_d_*} in *V_i_*, re-assign to the clusters that have the second highest probabilities.
9. Return to Step 2.

Step 8 is an extra process for obtaining fine-tuned clusters. For each cluster, a subset *D_s _*is chosen for the documents that have the lowest {*pz_d_*}s. If the selected subset is large, this step shuffles current clusters more. If it is too low, this procedure does not help get to the optimum at all. This step is similar to the mutation operation in genetic algorithms. Hence, the best strategy for this procedure is the high-rate subset selection for initial stages and eventually lowering the rate for later stages. However, for experiments, we select the fixed lower 1% documents in all iterations. Even though our method starts from random clusters, this shuffling process is helpful for achieving higher *α *scores in fewer trials.

*pr_d _*is the prior probability that affects the probability *pz_d _*(Eqn. 6 and 8). However, we generally have no clue which documents should or should not be included in specific themes. Thus, in the experiments, we set *pr_d _*to 0.5 so that it has no influence in computing *score_d _*(Eqn. 8). If one wants to assign some documents to a specific theme, it can be controlled by setting the value of *pr_d _*close to 1.

Another interesting feature of this algorithm is that *K *does not indicate the fixed number of clusters as output. Even though *K *is given as an initial number of clusters, it dynamically handles *K *by probabilistic evidence from documents. Since the proposed method assigns documents to clusters solely based on the highest *pz_d_*s, some cluster may disappear if it has relatively weak probabilities compared to others. If *K *is close to the number of documents, it gives more freedom to thematic clustering, but with increased processing time. If *K *is too small, the clustering time will be minimized, but extracted themes may be not be satisfactory. Therefore, we take care to set a reasonable maximum number *K *for the MEDLINE experiments.

## Results and discussion

### Experimental setup

For experiments, we use the 20-Newsgroup collection [15] for performance comparisons and the MEDLINE dataset [6] for theme extraction performance of the proposed method. The 20-Newsgroup collection consists of messages collected from 20 different Usenet newsgroups. Three subsets from the original corpus were used for our experiments [17]. Each subset has 100 messages randomly selected from each topic. News-Different-3 contains 300 messages from different topics on alt.atheism, rec.sport.baseball, and sci.space. News-Similar-3 contains 300 messages from similar topics on comp.graphics. comp.os.ms-windows, and comp.windows.x. News-Moderated-6 contains 600 messages from the topics, rec.sport.baseball, sci.space, alt.atheism, talk.politics.guns, comp.windows.x, and soc.religion.christian.

The MEDLINE dataset includes two subsets, Parkinson's Disease and Huntington's Disease. The document list for each disease is retrieved through PubMed^®^, and the documents including both title and abstract were gathered from the version of MEDLINE June 20, 2011. The Parkinson's Disease set consists of 25,992 documents obtained from a PubMed search with the query string, "Parkinson's disease". The Huntington's Disease set includes 5,602 documents obtained from a PubMed search with the query string, "Huntington's disease". While the Newsgroup datasets have gold standard clusters corresponding to the topics, MEDLINE datasets do not have any known answers. Hence, they are used for showing the stability of the proposed method and examples of extracted themes. Table 2 summarizes the datasets used for the experiments.

**Table 2:** Datasets used for the experiments

Datasets	Number of Documents	Number of Clusters
News-Different-3	300	3
News-Similar-3	300	3
News-Moderated-6	600	6

Parkinson's Disease	25992	-
Huntington's Disease	5602	-

News-Different-3, News-Similar-3, and News-Moderated-6 are from the 20-Newsgroup collection. Parkinson's Disease and Huntington's Disease are from the MEDLINE dataset.

All the datasets are pre-processed by removing stopwords and for the term set *T*, unigrams and bigrams are used as terms. The input parameters used for generating themes are 100 for *n_U _*and 0.5 for *pr_d_*. The initial number of clusters *K *is set to either 3 or 6 for the Newsgroup sets and 50 for the MEDLINE sets.

### Evaluation measure

We use two evaluation metrics for performance comparison and theme extraction. The normalized mutual information (NMI) [18] is a measure to evaluate the quality of clustering results. NMI is computed as follows [19]:

(13) $$NMI=\frac{\sum_{h,l}m_{h,l}\text{log}\left(\frac{m\cdot m_{h,l}}{m_{h}c_{l}}\right)}{\sqrt{\left(\sum_{h}m_{h}\text{log}\left(\frac{m_{h}}{m}\right)\right)\left(\sum_{l}c_{l}\text{log}\left(\frac{c_{l}}{m}\right)\right)}},$$

where *m *is the number of documents, *m_h _*is the number of documents in predicted cluster *h*, *c_l _*is the number of documents in answer cluster *l *and *m*_*h*,*l *_is the number of documents in both *h *and *l*. The NMI score is 1 when a cluster result perfectly matches the answer.

In addition, an F-score is defined to compare *n_U _*subject terms obtained from different runs. For the newsgroup sets, paired F-score evaluation [20] is used because the topics included are explicit and the number of clusters is also small. From one clustering result, we generate $\left(\begin{array}{c} n_{U}\\ 2 \end{array}\right)$ instance pairs for each cluster, where *n_U _*is the number of subject terms corresponding to each *V_i_*. Similarly, instance pairs are generated from the other clustering result. By doing this, precision can be defined as the number of common pairs between two sets divided by the number of pairs in one result. Recall can be defined as the number of common pairs between two sets divided by the number of pairs in the other. Finally, the paired F-score is the harmonic mean of precision and recall.

For MEDLINE datasets, the number of topics is less well defined and different views can be observed depending on statistical variation. Thus, instead of using the paired F-score defined above, we perform a F-score evaluation for the MEDLINE sets. For each cluster, i.e., theme, a title is chosen based on subject terms and its document set. The titles from two different sets are the elements for precision and recall evaluation. The F-score is simply the harmonic mean of precision and recall. How to choose a title of a theme is described in a later subsection.

### Clustering performance

The proposed method partitions documents based on themes, hence the clustering is basically to find a solution maximizing *α_t_'*s for each cluster. We have found empirically that the best clusterings come from the greatest total sum of squares from each cluster's *α *values, i.e., the theme score of a clustering result can be evaluated by

(14) $$Q=\overset{K}{\underset{i=1}{\sum }}{\left(\overset{n_{U}}{\underset{t=1}{\sum }}\alpha_{t}^{V_{i}}\right)}^{2},$$

where $\alpha_{t}^{V_{i}}$ is the value of *α*_*t *_in a cluster *V_i_*.

**Figure 1:**
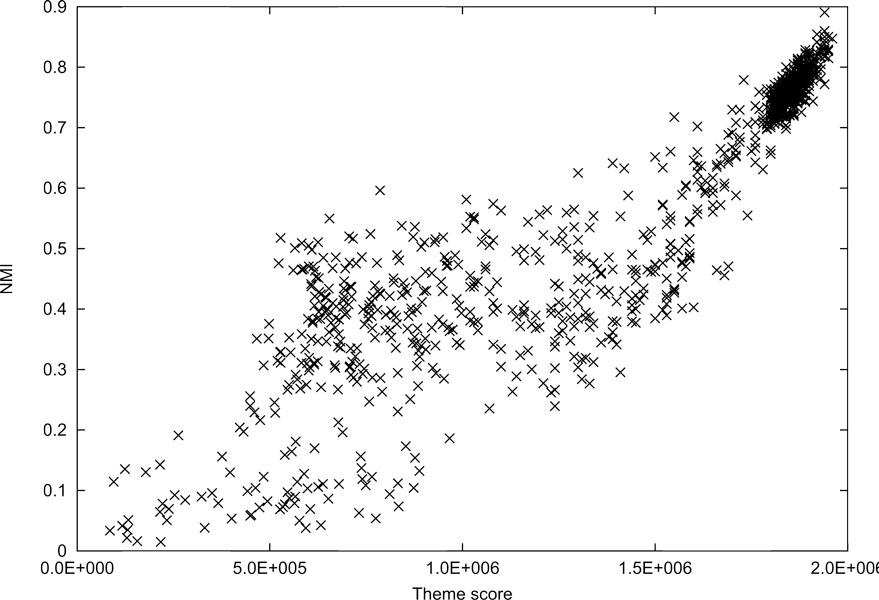
**Comparisons of theme scores and normalized mutual information (NMI) scores on News-Different-3**. This graph shows the correlation between theme scores and NMI scores on News-Different-3. The points are the clustering results obtained from 1,000 runs. The correlation coefficient is 0.904070.

**Figure 2:**
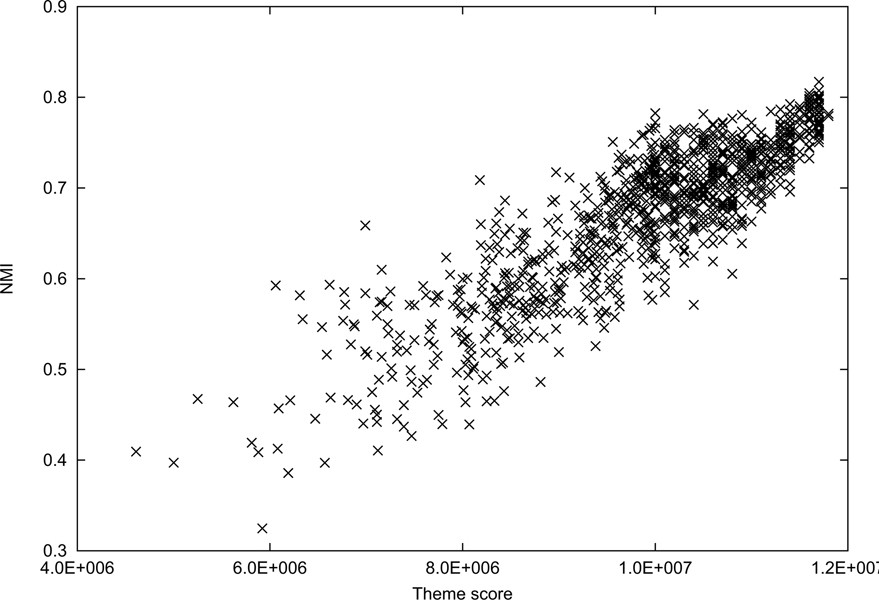
**Comparisons of theme scores and normalized mutual information (NMI) scores on News-Moderated-6**. This graph shows the correlation between theme scores and NMI scores on News-Moderated-6. The points are the clustering results obtained from 1,000 runs. The correlation coefficient is 0.871111.

A measure defined for clustering performance in this paper is NMI, however the theme score in (14) is not theoretically related to the NMI score. Figures 1 and 2 depict the correlation between theme scores and NMI scores on News-Different-3 and News-Moderated-6, respectively. In each graph, the points are the clustering results obtained from 1,000 runs. For low theme scores, there is some inconsistency with NMI. But, high theme scores clearly reach high NMI values. Even though the theme and NMI scores are not tightly coupled, it is evident that these scores are correlated in some way.

Table 3 shows the clustering performance for the proposed method (THEME) and other state-of-the-art approaches [17]. For THEME, the clustering results with the best theme score were chosen among 1,000 runs. DPMFS (Dirichlet process mixture with feature selection) [17] handles both document clustering and feature selection using a Dirichlet process mixture model and Gibbs sampling algorithms. DPMFS previously showed a competitive performance compared to EDCM and EM-MN on the 20-Newsgroup collection. EDCM (exponential-family approximation of the Dirichlet compound multinomial distribution) [21] performs document clustering using a mixture of EDCM with EM learning. EM-MN (multinomial mixture model with EM process) [22] is a standard clustering algorithm using a multinomial mixture model and EM process. These approaches are used for comparison because they provide the latest clustering performance on the Newsgroup set. Also, they are established on the popular frameworks such as Dirichlet process and EM-based learning.

**Table 3:** Performance comparison of THEME, DPMFS, EDCM, and EM-MN on the 20-Newsgroup collection

	THEME	DPMFS	EDCM	EM-MN
News-Different-3	0.847	0.688	0.734	0.867
News-Similar-3	0.103	0.231	0.163	0.081
News-Moderated-6	0.782	0.663	0.531	0.562

THEME, DPMFS, EDCM, and EM-MN are the proposed clustering method, a Dirichlet process mixture model, a Dirichlet compound multinomial model, and an EM-based mixture model, respectively.

In Table 3, THEME significantly outperforms other methods for News-Moderated-6. For News-Different-3, our method is still competitive to EM-MN, and outperforms DPMFS and EDCM. In News-Similar-3, all clustering methods show poor performance. Even though DPMFS produces the best score, it is not very meaningful because documents are still disorganized with that score. In our analysis, we find that terms in News-Similar-3 are not distinctive enough to identify clusters. Note that the current algorithm takes less than 3 minutes to finish all 1,000 runs, and the best score presented in the table can actually be reproduced with many fewer iterations. However, for the topic modeling approach LDA (Latent Dirichlet Allocation) [23] applied to these same datasets, the topic extraction time varies from 1 to 3 minutes for each run.

### Consistency of thematic clustering results

The proposed method is motivated by the idea that a set of documents has a theme or themes inside and a theme can be represented by its context, i.e. subject terms. From the previous section, it is shown that the theme approach produces a very competitive performance to state-of-the-art algorithms even though the theme concept has a weak link to common clustering approaches. However, due to the dynamic nature from the random start to the theme method, the clustering results may vary. Hence, we performed a stability test for best runs by F-scores. Each best run is the result with the best theme score among 500 runs.

Table 4 presents the average paired F-scores for the three best runs on the 20-Newsgroup dataset. The best runs on News-Different-3 and News-Moderated-6 include about 90% common term-pairs in clusters. One concludes that different best runs produce almost the same themes on the Newsgroup sets. News-Similar-3 is expected to have low common term-pairs since the clustering performance already shows low scores for all clustering approaches.

**Table 4:** Average paired F-scores from three best runs on the 20-Newsgroup collection

	F-score
News-Different-3	0.9387
News-Similar-3	0.3023
News-Moderated-6	0.8646

Three best runs on the 20-Newsgroup collection are compared using paired F-scores. Each best run is the result with the best theme score among 500 runs.

The theme method proposed in this paper produces a set of subject terms for each cluster. This quality helps humans understand the topics, which is particularly necessary for biomedical literature (See Table 5). For instance, the extracted terms can support easier browsing by grouping documents or summarizing document contents in PubMed. To see the effectiveness of biomedical literature clustering, we created two disease document sets, Parkinson's Disease and Huntington's Disease. Since themes computed on large document collections are more variable, it can be difficult to study them. Thus, unlike the 20-Newsgroup collection, we use a title extraction strategy to compare different best runs. We emphasize that a title is not necessarily a subject term and is used for the stability test only. To select a title for each cluster, all noun phrases from documents in the cluster are considered as title candidates. Title scores are then evaluated by multiplying document frequencies and *α *values for subject terms included in the noun phrases, i.e. the score *T *for the noun phrase *P *is defined as

(15) $$T\left(P\right)=\underset{t\subseteq P}{\sum }\alpha_{t}\cdot DF\left(t\right),$$

where *DF *(*t*) is the document frequency of the theme term *t*. Finally, the noun phrase with the highest score *T *is selected as the cluster title.

**Table 5:** An example for Parkinson's disease clusters

Subject terms	Titles
synuclein	alpha-synuclein
alpha synuclein	
alpha	
protein	
aggregation	

deep brain	deep brain stimulation
deep	
stimulation	
brain stimulation	
subthalamic	

lewy	lewy bodies
lewy bodies	
bodies	
lewy body	
dementia	

monoamine oxidase	monoamine oxidase
oxidase	
monoamine	
mao	
b	

mitochondrial	oxidative stress
oxidative	
complex i	
oxidative stress	
stress	

For each cluster, top 5 subject terms are listed. Titles are from these subject terms and the documents included in each cluster.

Table 6 shows the average F-scores for selected titles from the three best runs on the MEDLINE datasets. Both sets have more than 60% common titles for clusters. This means strong themes appeared consistently in each run. Weak themes tended to vary in different runs. This is inevitable under our assumption that a document has a unique theme. However, one cannot say weak themes are incorrect because they may also be a valid point of view for a given dataset.

**Table 6:** Average F-scores from three best runs on the MEDLINE data

	F-score
Parkinson's Disease	0.6572
Huntington's Disease	0.6308

F-score results of three best runs on each MEDLINE dataset are averaged. A best run is the result with the best theme score among 500 runs.

### Theme extraction on biomedical literature

MEDLINE is a promising source where the thematic clustering algorithm can be utilized. However, there is a limit to evaluating how well clustering is done on this MEDLINE data because no gold standard is available. MeSH (Medical Subject Headings) is a controlled vocabulary for indexing and searching biomedical literature [24]. MeSH terms are organized in a hierarchical structure and are used to indicate the topics of an article. Thus, these MeSH terms can be helpful to identify how well a set of documents are grouped by topic. For MeSH terms which appear repeatedly in a cluster, *p*-values can be calculated using the hypergeometric distribution [25]. If documents are randomly clustered, MeSH terms in the document set will have high (meaningless) *p*-values. If there are MeSH terms with low *p*-values, this means that the cluster is formed to include the corresponding topics.

Table 7 presents the average number of clusters and *p*-values for thematic clusters on the MEDLINE datasets. Each best run is the result with the best theme score among 500 runs, and three best runs are used to build this table. *P*-values shown in the table are the averages over the most significant 10 MeSH terms obtained from each cluster. For Parkinson's and Huntington's sets, the average *p*-values are 2.56E-10 and 4.11E-11, respectively. This indicates that clustering results are not random, and the clusters tend to partition by humanly recognized subjects. It is also interesting to see that the average number of clusters is 46 and 21.5 on Parkinson's and Huntington's sets, respectively. Starting from 50 clusters, Parkinson's Disease ended up with 46 clusters on average. Huntington's Disease ended up with 21.5 clusters on average. As mentioned in Methods, the proposed algorithm handles a set of documents dynamically. This results in a smaller number of clusters when duplicate themes are consolidated during a learning step.

**Table 7:** Analysis of three best runs on the MEDLINE data

	Number of clusters	*p*-value
Parkinson's Disease	46.0	2.56E-10
Huntington's Disease	21.5	4.11E-11

For each MEDLINE dataset, clustering was performed 500 times, and the best run was selected. The number of clusters and the average of *p*-values of the 10 strongest MeSH terms in each cluster were recorded. This was repeated three times, and averages of the resulting values are given in this table.

Table 5 is an example of the cluster results from the Parkinson's Disease set. Five clusters are listed with their top 5 subject terms (themes) and their titles. Alpha-synuclein is a protein that plays a role in development of Parkinson's disease [26]. Deep brain simulation is a surgical treatment for various neurological symptoms seen in Parkinson's disease [27]. Lewy bodies are hallmark lesions of degenerating neurons, and these lesions are diagnostic for Parkinson's disease [28]. Monoamine oxidase type B inhibitors are an antidepressant drug for the treatment of Parkinson's disease [29]. Oxidative stress contributes to the cascade leading to dopamine cell degeneration in Parkinson's disease [30]. We find that the proposed method performs well in extracting concepts used in text documents. In addition, the selected subject terms and the title are helpful in understanding the themes.

## Conclusions

We proposed a clustering algorithm based on thematic analysis of text documents. Unlike common clustering approaches, the proposed method focuses on themes that are implicitly described in text. Given documents, a set of subject terms are selected and used for clustering documents in a probabilistic framework through an EM algorithm. Applied to the 20-Newsgroup collection and the MEDLINE dataset, our theme method has a competitive performance compared to other state-of-the-art clustering approaches. Also, the extracted terms and the title selection strategy show that the proposed method effectively captures sub-topics of a set of text documents.

The theme-based approach only utilizes a limited set of terms for clustering, however clustering performance matches that of the best performing algorithms. This indicates that extracted subject terms are an effective summary version of clusters. In particular, the explanatory feature of the theme algorithm is distinctive. This can be useful when human understanding is required. The biomedical domain benefits from this understanding. A search query can return a large set of documents including multiple biological topics. The theme-based clustering helps organize these documents by content. Particularly, it provides a set of terms that describe the organized documents. This process enables more focused searching and a better browsing experience.

Future study includes a systematic approach to finding good initial clusters. Currently, initial clusters are randomly generated, and the result showing the best theme score is chosen among multiple trials. Even though clustering time is reasonably fast, it is still too slow to apply to all of MEDLINE. Finding better seed clusters or parallelizing theme generation processes will shorten clustering time, and increase the chance of obtaining optimal solutions.

## Competing interests

The authors declare that they have no competing interests.

## Authors' contributions

SK and JW proposed the idea and SK carried out the computational experiments and analysis. JW supervised the project and revised this manuscript. All authors read and approved the final manuscript.
